# The Diagnostic Performance of Afirma Gene Expression Classifier for the Indeterminate Thyroid Nodules: A Meta-Analysis

**DOI:** 10.1155/2019/7150527

**Published:** 2019-08-20

**Authors:** Ying Liu, Bihui Pan, Li Xu, Da Fang, Xianghua Ma, Hui Lu

**Affiliations:** ^1^Department of Endocrinology, The First Affiliated Hospital of Nanjing Medical University, 300 Guangzhou Road, Nanjing 210029, China; ^2^Department of Hematology, The First Affiliated Hospital of Nanjing Medical University, 300 Guangzhou Road, Nanjing 210029, China; ^3^Department of Nutriology, The First Affiliated Hospital of Nanjing Medical University, 300 Guangzhou Road, Nanjing 210029, China; ^4^Department of General Surgery, The First Affiliated Hospital of Nanjing Medical University, 300 Guangzhou Road, Nanjing 210029, China

## Abstract

**Background:**

Approximately 15 to 30% of thyroid nodules evaluated by fine-needle aspiration (FNA) were classified as indeterminate; the accurate diagnostic molecular tests of these nodules remain a challenge. We aimed to evaluate the diagnostic performance of Afirma gene expression classifier (GEC) for the indeterminate thyroid nodules (ITNs).

**Methods:**

Studies published from January 2005 to December 2018 were systematically reviewed. The gold reference standard relied on the histopathologic results diagnosis from thyroidectomy surgical specimens. MetaDisc software was used to investigate the pooled sensitivity, specificity, negative predictive value (NPV), positive predictive value (PPV), diagnostic odds ratio (DOR), and summary receiver operating characteristic (SROC) curves.

**Results:**

A total of 18 studies involving 5290 patients with 3290 cases of ITNs were included. Collected data revealed that the pooled sensitivity of GEC was 95.5% (95% CI 93.3%–97.0%, p < 0.001), the specificity was 22.1% (95% CI 19.4%-24.9%, p < 0.001), the NPV was 88.2% (95% CI 0.833–0.921, p < 0.001), the PPV was 44.3% (95% CI 0.416–0.471, p < 0.001), and the DOR was 5.25 (95% CI 3.42–8.04, p= 0.855).

**Conclusion:**

The GEC has quite high sensitivity of 95.5% but low specificity of 22.1%. The high sensitivity makes it probable to rule out malignant nodules. Thus, over half of nodules with GEC-suspicious results still require further validation like molecular markers, diagnostic surgery, or long follow-up, which limits its use in future clinical practice.

## 1. Introduction

Approximately 15 to 30% of thyroid nodules evaluated by fine-needle aspiration (FNA) are classified as indeterminate, including atypia of undetermined significance/follicular lesion of undetermined significance (AUS/FLUS, category III), follicular neoplasm or suspicious for follicular neoplasm (FN/SFN, category IV), and suspicious for malignancy (SM, category V) according to the Bethesda System for Reporting Thyroid Cytopathology (TBSRTC) [1]. The present guidelines recommend repeated FNA for category III lesions, lobectomy for category IV lesions, and repeated category III lesions [2–4]. However, the malignancy risk in TBSRTC categories III and IV ranges between 5% and 30% after surgery [5]. Patients with cytological ITNs are often referred for diagnostic surgery, though most of these nodules finally prove to be benign [6]. The Afirma gene expression classifier (GEC) measures the expression of 167 gene transcripts to determine whether the nodules are benign or malignant [7]. In 2012, a prospective, multicenter validation trial of the Afirma GEC involving 265 ITNs demonstrated a sensitivity of 92% and a specificity of 52% in TBSRTC III/IV nodules [7]. In the last decade, some studies [8–10] have evaluated its effects on ITNs but the results were inconsistent, probably due to the rates of indeterminate biopsy result varying among the hospitals and tertiary centers [11]. In 2016, a meta-analysis including seven studies of GEC revealed the pooled sensitivity of 95.7% and the specificity of 30.5% and concluded it as a rule-out malignancy test [9]. However, Sacks et al. demonstrated that there were no significant changes in surgery rates and malignant prevalence by comparing pre-Afirma and post-Afirma cases [12]. We checked the database and included 18 newly published studies to provide a more comprehensive analysis on the diagnostic performance of GEC and discuss its role in decision-making process of thyroid surgery.

## 2. Methods

### 2.1. Data Sources and Search

We searched PubMed and Embase for studies published between January 2005 and December 2018. We also checked the Cochrane Library with the same keywords. A total of 126 studies were identified. After excluding duplicates, reviews, commentary, insufficient data, 18 studies [7, 12–28] examined the performance of GEC for ITNs. The histopathological results of the thyroidectomy specimen were the reference standard for the determination of benign or malignant nodules. We used a QUADAS-2 report [29] for the included studies to assess the bias and applicability of the test.

In PubMed database, the keywords were a combination of “Thyroid Nodule/diagnosis”[Majr] OR “Thyroid Nodule/pathology”[Majr] OR “Thyroid Nodule/surgery”[Majr] AND “gene expression classifier” OR “GEC”. Embase search was done using the following keywords (“Thyroid Nodule/diagnosis”[Mesh] OR “Thyroid Nodule/surgery”[Mesh]) AND (“gene expression classifier” OR “GEC”). Meanwhile, we checked the references of included literatures to identify additional relevant publications.

### 2.2. Study Selection and Eligibility Criteria

#### 2.2.1. Inclusion Criteria for Studies

1. Indeterminate thyroid results via FNA that included categories III, IV, and V.
2. Use of Afirma GEC test as an index test.
3. Histopathologic results diagnosis from thyroidectomy surgical specimens as gold reference standard.
4. Incidental microcarcinomas were not included in the analysis.

#### 2.2.2. Exclusion Criteria for Studies

1. Opinions, reviews, commentary, case reports, and insufficient data.
2. Lack of clinical characteristics of nodules, clear inclusion, and exclusion criteria.
3. Absence of surgical histopathology results.

**Figure 1:**
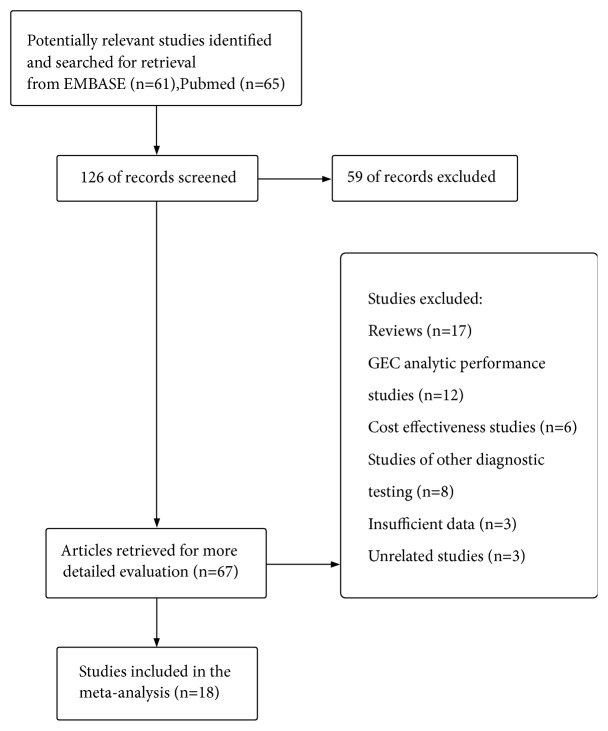
Flowchart showing algorithm for screening and study selection.

We screened the studies following the process that was illustrated in Figure 1. A total of 18 studies met the inclusion criteria via the evaluation of QUADAS-2 questionnaire [29].

### 2.3. Data Extraction

Two authors were engaged in reviewing the literatures from PubMed database and Embase independently according to the inclusion criteria. All conflicts were resolved through consensus within the groups. A third reviewer assessed all the discrepant items and the major opinion was used to resolve the disagreement between the reviewers.

### 2.4. Statistical Analysis

The present work followed the structure of the PRISMA statement. Analyses were conducted using MetaDisc 1.4. We calculated pooled sensitivity, specificity, DOR, SROC, and the prediction ellipses for the hierarchical ordinal regression for ROC curves (HROC) model. We also used Cochrane Review Manager Version 5.3 (RevMan; 2014) to perform risk of bias evaluations of studies included in this meta-analysis. Deek's funnel plot asymmetry test was adopted as the way of evaluating publication bias both in each section of the analysis.

## 3. Results

### 3.1. Performance of Gene Expression Classifier in ITNs

A total of 18 studies [7, 12–28] meeting eligibility criteria were included in this meta-analysis, main characteristics of all selected reports were showed in Table 1. We got 5290 patients assessed by FNA; 3390 nodules were categorized as ITNs, 2889 of which underwent gene expression classifier finally. Of the 2889 nodules with GEC results, 1187 (41.1%) were GEC-benign, 1599 (55.4%) were GEC-suspicious, and 101 (3.6%) were GEC-unsatisfactory. Of 1187 benign nodules, 228 (19.2%) benign nodules underwent surgery; 27 (11.8%) of them proved to be malignant while 201 (88.2%) were benign after thyroidectomy. Meanwhile, 1371 of 1599 nodules categorized as suspicious GEC results underwent surgery; 617 (45.0%) of them were malignant while 754 (55.0%) were benign. In 101 nodules with GEC-unsatisfactory results, 18 (3.6%) had surgery, 1 (5.6%) proved to be malignant while 17 (94.4%) were benign. Since not all studies included cytological subtypes performance of with GEC results, we calculated nodules with cytological subtypes (N=1628), which are given in Table 2. After underwent surgery, all samples were proved to be either benign or malignant.  Table 3 demonstrates the correlation between overall surgery follow-up and GEC results with available data. After surgical resection, the malignant call risk was 645/1617 (39.9%) while the benign call rate was 972/1617 (60.1%).

Several original articles missed part of the detailed pathological results of surgical samples, just described surgical results as benign or malignant. We collected the available pathological information.  Table 4 shows the surgical pathological diagnoses at resection of Afirma results with cytological subtypes (with as much as available data) (N=225).

The most surgical benign nodules were follicular adenoma, adenomatoid nodule, thyroiditis, etc. The most surgical malignant lesions are classic variant of papillary thyroid carcinomas (cvPTC) and follicular variant of papillary thyroid carcinomas (fvPTC). The summary of final histopathologic subtypes of all samples are available in Table 5 (N=960). The benign thyroid nodules proved by surgical resection are follicular adenoma, benign follicular nodule and adenomatoid nodule, etc. The most surgical malignant lesions are cvPTCs and fvPTCs.

### 3.2. Summary Estimates of Sensitivity, Specificity, NPV, PPV, DOR, and Summary ROC Curves

The analysis of diagnostic threshold revealed the spearman correlation coefficient was 0.414, p=0.111. We concluded that there was no threshold effect in this meta-analysis.

**Figure 2:**
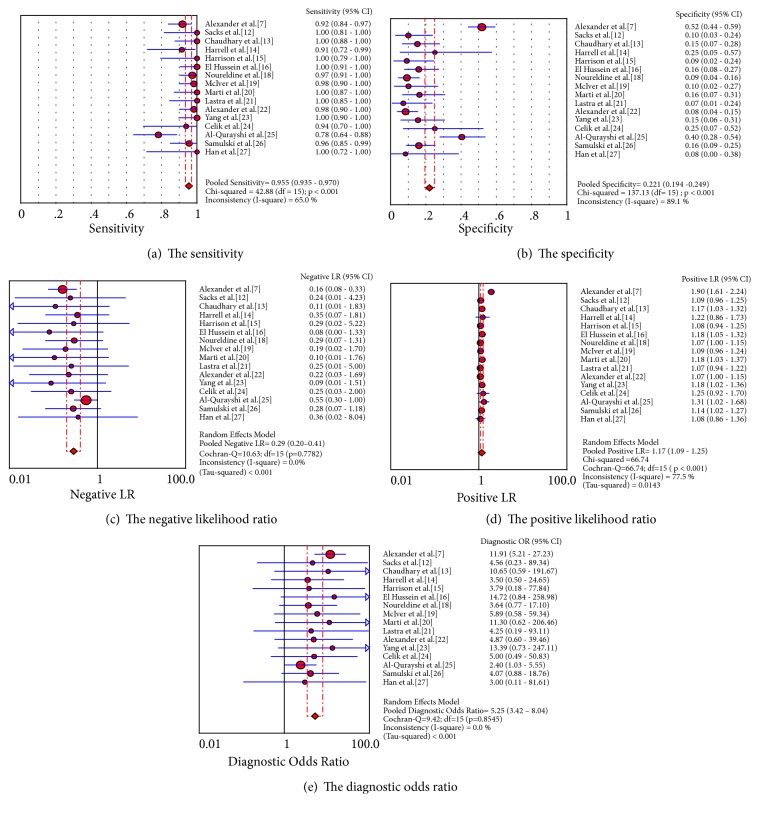
The pooled sensitivity, specificity, negative likelihood ratio, positive likelihood ratio, and diagnostic odds ratio of the analysis.

Table 6 shows the pooled sensitivity, specificity, confidence intervals and heterogeneity results of the test. The pooled sensitivity of GEC is 95.5% (95% CI 93.5%–97.0%, I^2^ value 65.0%, p < 0.001), the pooled specificity is 22.1% (95% CI 19.4%-24.9%, I^2^ value 89.1%, p < 0.001), the PLR is 1.167 (95% CI 1.088–1.252, I^2^ value 77.5%, p < 0.001), the NLR is 0.285 (95% CI 0.199–0.410, I^2^ value 0.00%, p= 0.778), the NPV is 88.2% (95% CI 0.833–0.921, I^2^ value 41.1%, p < 0.001), the PPV is 44.3% (95% CI 0.416–0.471, I^2^ value 65.0%, p < 0.001), and the DOR is 5.25 (95% CI 3.42–8.04, I^2^ value 0.00%, Q 9.42, p=0.855). The forest plots exhibit the pooled sensitivity, specificity, PLR, NLR, diagnostic score, and DOR (Figures 2(a)–2(e)). Since the false negative and true negative values of two included studies [17, 27] were 0, the original data of these two studies was dropped by the MetaDisc software.

Since the I^2^ values of the sensitivity, specificity, PLR, and NPV were more than 50%, we conducted the metaregression analysis (inverse variance weights) to investigate the sources of heterogeneity. The metaregression revealed whether the original GEC test studies were conducted in single or multiple centers was the main source of heterogeneity (p=0.032) (Table 7).

**Figure 3:**
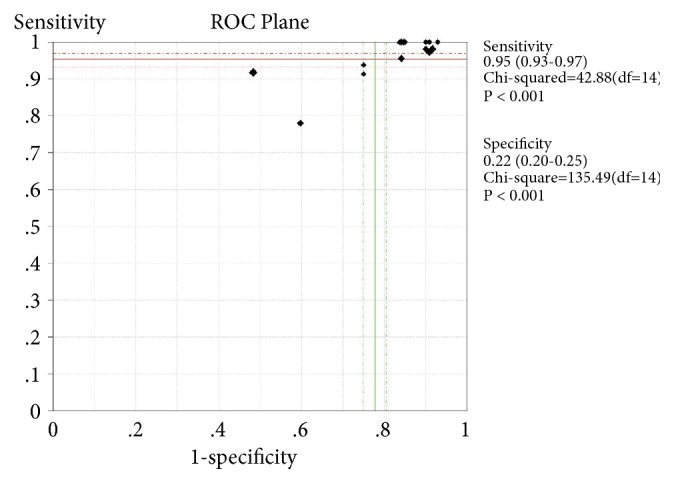
The pooled ROC plane of the analysis.

**Figure 4:**
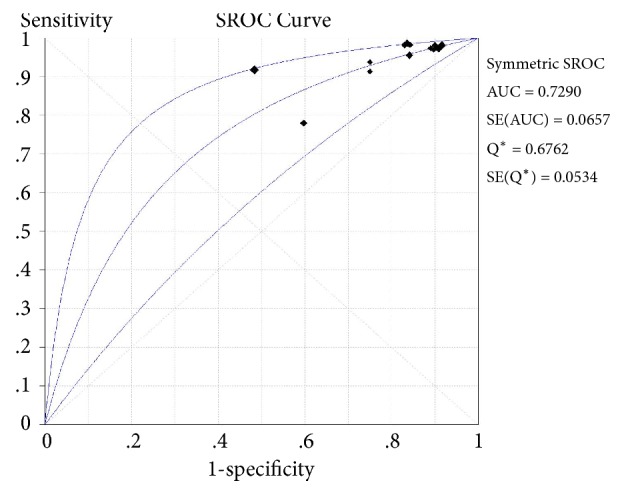
Summary receiver operating characteristic (SROC) curve with prediction and confidence contours.

**Figure 5:**
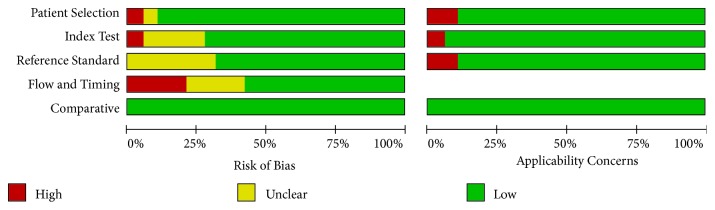
Risk of bias and applicability concerns: review authors' judgements about each domain presented as percentages across the included studies.

The bivariate logistic regression is described in Table 8. The ROC plane is in Figure 3. The SROC curve has been shown in Figure 4 with prediction and confidence contours. The area under the curve (AUC) is 0.73. The evaluation of bias in this meta-analysis is in Figure 5.

### 3.3. Publication Bias

**Figure 6:**
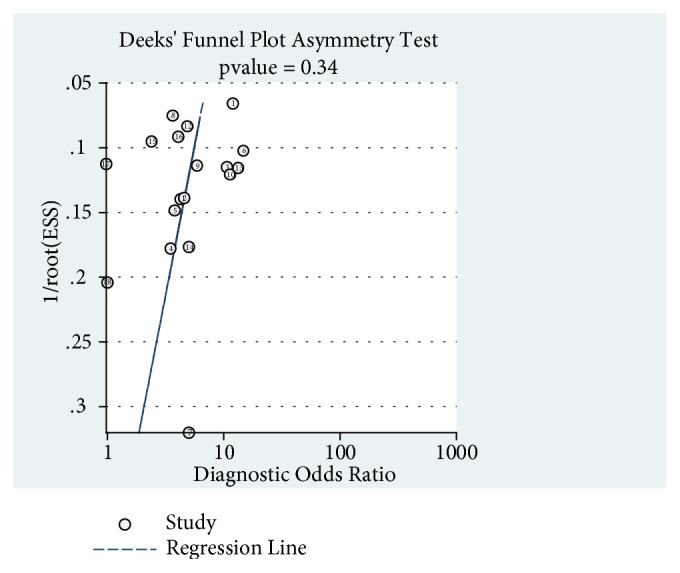
Deek's funnel plot asymmetry test of the analysis.

We conducted Deek's funnel plot asymmetry test to evaluate publication bias in each section of the analysis (Figure 6). As the p-value is 0.34, we concluded that no obvious publication bias was found in every section of this meta-analysis.

## 4. Discussion

Thyroid cytopathological ITNs are usually referred to thyroidectomy or lobectomy and up to 74% of patients with cytologically indeterminate nodules are operated [5]. To some extent, ultrasound-guided FNA with on-site cytopathology improves both adequacy and accuracy of preoperative diagnoses in ITNs.

The Afirma GEC developed by Veracyte (South San Francisco, CA) measures 167-gene mRNA expression panel of thyroid nodules to distinguish benign and malignant nodules. Since commercially available in 2011, the test has significantly prevented avoidable thyroid surgeries. Most studies regarded it as a tool to rule out malignant lesions and potential for risk assessment [7, 9].

A systematic review [30] which evaluated the methods of studies of GEC and concluded the most common methodologic drawback was lack of reference standard diagnosis analyses to unexcised ITNs with GEC-benign results, which resulted in overestimating the specificity. The performance of GEC could range widely between tertiary care facilities and comprehensive hospitals [9]. Patients' selection for surgery may affect both accuracy and clinical applicability of the test. Noureldine et al. proposed a surgical management algorithm and found that GEC did not change the surgical decision-making process significantly [18]. After long follow-up period, there were no significant malignancy differences between the two groups [31].

One earlier meta-analysis [9] assessed the performance of GEC. By adding newly published studies of GEC in recent years and pathological results after surgery, our results revealed that the GEC's sensitivity was 95.4%, the specificity was 22.3%. The diagnostic profiling of GEC is mainly limited to papillary and follicular thyroid carcinoma partly due to the relatively low prevalence of medullary and anaplastic thyroid cancer. Our present data revealed that the pooled NPV of GEC was not as high as previous studies [7, 9, 32].

The present study summarized the final pathological outcomes of GEC nodules after surgery. The high sensitivity and NPV make GEC as an effective approach to rule out malignant lesions in thyroid nodules with an indeterminate cytology. Taking the pooled postoperative pathological data into consideration, most GEC-suspicious nodules with benign pathological results after surgery are follicular adenomas (31.2%), benign follicular nodules (15.6%) and adenomatoid nodules (13.0%). The adenomatoid nodule is featured as a densely cellular follicular proliferation lack of capsule in histology. In the TBSRTC, the adenomatoid nodule is divided into category III or category IV [1]. According to a study of 234 thyroid FNA, the adenomatoid nodules were easily incorrectly diagnosed as follicular neoplasms [33]. Chronic thyroid inflammation is commonly regarded as chronic lymphocytic thyroiditis (CLT), characterized with diffuse lymphocytic infiltration in the thyroid glands. The impact of CLT on clinical and pathological outcomes of DTC remains unknown [34]. Some studies supported that DTC patients with CLT had a better prognostic outcome compared with those without CLT [35]. Most nodules with benign pathological results and well-differentiated PTC are proliferated from thyroid follicular cells. Benign nodules include follicular carcinoma and oncocytic adenoma. According to Table 5, follicular adenoma is the most common benign thyroid lesions (31.2%); the second most common is benign follicular nodules (16.0%). Malignant lesions such as cvPTC (44.3%) and fvPTC (38.3%) are classified into well-differentiated PTC.

An individual study [36] demonstrated that a predominance of Hürthle cells group led to an increased rate of suspicious GEC results with lower malignant risk than AUS/FLUS or FN/SFN nodules. HCNs partly contributed to the false positive rate of GEC. Considering the recent reclassification of the encapsulated fvPTC as “noninvasive follicular neoplasm with papillary-like nuclear features (NIFTP)”, prior studies seldom reclassified fvPTC as NIFTP, which could give rise to unreliable estimates of cancer prevalence and PPV [37]. However, only limited data is available to evaluate the accuracy of GEC in HCN or NIFTP cases.

The Thyroid Imaging Reporting and Data System (TI-RADS) was designed to quantify malignancy of thyroid nodes [38, 39]. It was based on suspicious ultrasound features such as solid component, hypoechogenicity or marked hypoechogenicity, irregular margins, microcalcifications or mixed calcifications, and taller-than-wide shape. Gathered data of thyroid nodes showed the sensitivity of TI-RADS was 97.4–99.1% and the NPV was 98.1-99.1% [40, 41]. The TI-RADS and American Thyroid Association (ATA) guideline have greatly help physicians stratify the malignancy risk of ITNs. Recently, molecular tests with higher accuracy, together with TI-RADS, were applied for ITNs to decrease the false positive rates.

The BRAF^V600E^ mutation is detected in more than half of papillary thyroid cancer. BRAF mutation has low prevalence in the FN/SFN and AUS/FLUS while high in the SM cytology thyroid lesions [42, 43]. However, adding the BRAF V600E mutation to GEC did not improved the diagnostic sensitivity and specificity [44].

The next-generation sequencing panel, ThyroSeq v2, detected 14 cancer gene mutations with more than 1000 hotspots and 42 types of gene fusions or rearrangements in thyroid cancer [45]. A meta-analysis evaluated GEC from 1086 nodules and ThyroSeq v2 from 459 nodules to assess the preoperative diagnostic accuracy of ITNs [46]. Pooled data showed the sensitivity was 98% and 84%, and the specificity was 12% and 78%, respectively. In this meta-analysis, the pooled sensitivity of GEC was higher than our analysis while the pooled specificity was lower than our analysis. Therefore, the superiority of the GEC test lies in ruling-out of malignancy (higher sensitivity) and the ThyroSeq is a better test of ‘ruling-in' thyroid neoplasm (higher specificity).

The risk of malignancy in ITNs was nearly 38.6% in our analysis, indicating that over half patients had underwent undue surgeries and conservative approaches could be considered for ITNs. The final decision of a diagnostic surgery or follow-up depends on US features, histological characteristics, and molecular test results.

## 5. Conclusions

The present meta-analysis has summarized the previously reported performance of GEC. We regard GEC as an effective approach to rule out malignant lesions in ITNs. Since the most benign nodules with GEC-suspicious results are follicular adenomas, benign follicular nodules and adenomatoid nodules, it is essential to combine other molecular markers to improve the specificity of GEC. The probability of malignancy and clinical management of nodules with GEC-suspicious still needs further investigation.

## 6. Limitations

Our study has several limitations. First, we failed to obtain the pathologic diagnosis of all the resected nodules, due to the missed original contents in some of the included studies. Second, it is not sure if there were geographic, race, and region variations regarding the GEC results and none of which mentioned the race of participants. Finally, some of the included studies lack the information of long-term follow-up for GEC-benign nodules or when the nodules underwent FNA during follow-up.

## Figures and Tables

**Table 1:** Summary of the studies' characteristics included in the analysis.

First Author	Research Method	Center	Study Time	Site of GEC	Included Nodules
Alexander et al.[7]	P	Multi Centers	2011.1-2012.8	Veracyte Inc	567
Sacks et al.[12]	R	Single Center	2012.1-2014.12	tertiary	567
Chaudhary et al.[13]	R	Single Center	2009.7-2015.1	Veracyte Inc	158
Harrell et al.[14]	U	Single Center	2011.1-2013.4	Veracyte Inc	58
Harrison et al.[15]	R	Single Center	2013.8-2015.3	Veracyte Inc	115
El Hussein et al.[16]	R	Single Center	2012.1-2016.7	Veracyte Inc	227
Abeykoon et al.[17]	R	Single Center	2014.12-2015.2	Veracyte Inc	34
Noureldine et al.[18]	R	Single Center	2012.1-2014.12	Veracyte Inc	273
McIver et al.[19]	P	Multi Centers	2011.5-2012.12	Veracyte Inc	105
Marti et al.[20]	R	Multi Centers	2013.2-2014.12	Veracyte/tertiary	165
Lastra et al.[21]	R	Single Center	2011.2-2014.1	Veracyte Inc	132
Alexander et al.[22]	R	Multi Centers	2010.9-2013.1	Veracyte Inc	339
Yang et al.[23]	R	Single Center	2012.8-2014.4	Veracyte Inc	187
Celik et al.[24]	P	Single Center	2011.12-2014.7	Veracyte Inc	66
Al-Qurayshi et al.[25]	R	Single Center	2013.1-2016.2	tertiary	154
Samulski et al.[26]	R	Single Center	2011.1-2015.12	U	294
Kay-Rivest et al.[27]	R	Multi Centers	2013.?-2015.?	Veracyte Inc	172
Han et al.[28]	R	Single Center	2009.8-2013.3	U	114

GEC, gene expression classifier; R: retrospective; P: prospective; U: unclear.

**Table 2:** Performance of cytological subtypes indeterminate cases with GEC results (N=1628).

GEC Results	AUS/FLUS	SFN	SM	Total
Benign	427(41.6%)	206(39.0%)	18(24.3%)	651
Suspicious	554(54.0%)	301(57.0%)	55(74.3%)	910
Unsatisfactory	45(4.4%)	21(4.0%)	1(1.4%)	67
Total	1026	528	74	1628

AUS/FLUS, atypia of undetermined significance or follicular lesion of undetermined significance; SFN, follicular or Hürthle cell neoplasm or suspicious for follicular neoplasm; SM, suspicious for malignancy.

**Table 3:** Correlation between overall surgical pathologically cases and GEC results (N=1617).

GEC Results	Surgery Follow-up Results		Total
	Benign	Malignant	

Benign	201(88.2%)	27(11.8%)	228
Suspicious	754(55.0%)	617(55.0%)	1371
Unsatisfactory	17(94.4%)	1(5.6%)	18
Total	972(60.1%)	645 (39.9%)	1617 (100.0%)

**Table 4:** Pathological diagnoses at resection of Afirma results with cytological subtypes (N=225).

		Pathological Diagnoses after Surgery			
		Benign		Malignant	
FNA Diagnosis	No.(%)	Diagnosis	No.(%)	Diagnosis	No.(%)

Bethesda III (AUS/FLUS)	139(61.8%)	Follicular adenoma	14(10.1%)	cvPTC	37(26.6%)
		Adenomatoid nodule	23(16.5%)	fvPTC	34(24.5%)
		Thyroiditis	7(5.0%)	PTC with HC	2(1.4%)
		Graves' Disease	2(1.4%)	feature	
		HCA	4(2.9%);	FTC	1(0.7%)
		Chronic inflammation	1(0.7%)	HCC	1(0.7%)
		Nodular hyperplasia	12(8.6%)	Others	1(0.7%)
		Total	63	Total	76

Bethesda IV (FN)	77(34.2%)	Follicular adenoma	10(13.0%)	cvPTC	25(28.6%)
		HCA	17(22.1%)	fvPTC	13(16.9%)
		Adenomatoid nodule	6(7.8%)	FTC	1(1.3%)
		Nodular hyperplasia	1(1.3%)	HCC	4(5.2%)
		Total	34	Total	43

Bethesda V (SM)	9(4.0%)	None		fvPTC	7(5.5%)
		Total	0	Others	2(1.6%)
				Total	9

All Categories	225(100.0%)		97		128

PTC, papillary thyroid carcinoma; FTC: follicular thyroid carcinoma; MTC: medullary thyroid cancer; AN, adenomatous/hyperplasic nodule; cvPTC, classic variant of papillary thyroid carcinoma; fvPTC, follicular variant of papillary thyroid carcinoma; NIFPTC, noninvasive follicular neoplasm with papillary-like nuclear features; PTC, HC feature; HCA, Hürthle cell adenoma; HCC, Hürthle cell carcinoma.

**Table 5:** Summary of final surgical pathology at resection of all surgical nodules (N=960).

	Pathological Diagnoses after Surgery			
	Benign		Malignant	
	Diagnosis	No.(%)	Diagnosis	No.(%)

Final Surgical Pathology	Follicular adenoma	139(31.2%)	cvPTC	228(44.3%)
	Benign follicular nodule	71(16.0%)	FvPTC	197(38.3%)
	Adenomatoid nodule	58(13.0%)	PTC, HC	
	Nodular hyperplasia	56(12.6%)	features/variant	2(0.4%)
	HCA	49(11.0%)	NIFPTC	15(2.9%)
	Oncocytic follicular		FTC	39(7.6%)
	adenoma	22(4.9%)	HCC	24(4.7%)
	Chronic lymphocytic		MTC	6(1.2%)
	thyroiditis	9(2.0%)	Malignant lymphoma	2(0.4%)
	MNG	5(1.1%)	Others	2(0.4%)
	Graves' Disease	2(0.4%)		
	Chronic inflammation	1(0.2%)		
	Others	33(7.4%)		

Total		445(100%)		515(100%)

PTC, papillary thyroid carcinoma; FTC: follicular thyroid carcinoma; MTC: medullary thyroid cancer; AN, adenomatous/hyperplasic nodule; cvPTC, classic variant of papillary thyroid carcinoma; fvPTC, follicular variant of papillary thyroid carcinoma; NIFPTC, noninvasive follicular neoplasm with papillary-like nuclear features; PTC, HC feature: papillary thyroid carcinoma with Hürthle cell; HCA, Hürthle cell adenoma; HCC, Hürthle cell carcinoma.

**Table 6:** Pooled sensitivities: confidence interval and heterogeneity results.

	Estimate	95% CI	I^2^	Q	P
Se	0.955	(0.935, 0.970)	65.00%	42.87	<0.001
Sp	0.221	(0.194, 0.249)	89.10%	137.13	<0.001
PLR	1.167	(1.088, 1.252)	77.50%	66.74	<0.001
NLR	0.285	(0.199, 0.410)	0.00%	10.63	0.778
DOR	5.248	(3.425, 8.043)	0.00%	9.42	0.855

Se, sensitivity; Sp, specificity; PLR, positive likelihood ratio; NLR, negative likelihood ratio; DOR, diagnostic odds ratio; I^2^, inconsistency I-square; Q, Chi-square.

**Table 7:** The metaregression of the test.

Model 1: the variables are method, site, center, and patients					
Variable	Coefficient	Standard Error	p - value	RDOR	95%CI

Cte.	1.23	1.3157	0.3719	----	----
S	0.109	0.1715	0.5384	----	----
Method	-0.253	0.4549	0.5901	0.78	(0.28;2.14)
Site	-0.08	0.5308	0.8838	0.92	(0.28;3.01)
Center	0.594	0.8687	0.5096	1.81	(0.26;12.55)
Patients	0.001	0.0021	0.6037	1	(1.00;1.01)

Model 2: the variables are method, center, and patients					

Variable	Coefficient	Standard Error	p - value	RDOR	95%CI

Cte.	1.09	0.9294	0.2655	----	----
S	0.127	0.1264	0.338	----	----
Method	-0.277	0.426	0.5289	0.76	(0.30;1.94)
Center	0.591	0.8685	0.51	1.81	(0.27;12.22)
Patients	0.001	0.0021	0.5692	1	(1.00;1.01)

Model 3: the variables are method and center					

Variable	Coefficient	Standard Error	p - value	RDOR	95%CI

Cte.	1.295	0.8614	0.1586	----	----
S	0.122	0.1262	0.3527	----	----
Method	-0.257	0.4246	0.5569	0.77	(0.31;1.95)
Center	0.998	0.5232	0.0806	2.71	(0.87;8.48)

Model 4: the variable is center					

Variable	Coefficient	Standard Error	p - value	RDOR	95%CI

Cte.	0.892	0.5458	0.126	----	----
S	0.11	0.1245	0.3949	----	----
Center	1.133	0.473	0.0323	3.11	(1.12;8.63)

RDOR: relative diagnostic odds ratios.

**Table 8:** The bivariate logistic regression of the test.

Bivariate parameter	Coefficient	Standard error	95% CI
E(logitSe)	4.016	0.482	(3.072,4.960)
E(logitSp)	-1.866	0.249	(-2.355, -1.377)
Var(logitSe)	1.373	0.865	(0.399, 4.722)
Var(logitSp)	0.766	0.352	(0.312, 1.884)
Corr(logits)	-0.898	0.114	(-0.989, -0.304)

Var: variable; Corr: correlation.

## Data Availability

The datasets used or analyzed during the current study are available from the corresponding authors on reasonable request.

## Abbreviations

NPV: Negative predictive value
PPV: Positive predictive value
DOR: Diagnostic odds ratio
SROC: Summary receiver operating characteristic
FNA: Fine-needle aspiration
AUS/FLUS: Atypia of undetermined significance/follicular lesion of undetermined significance
FN/SFN: Follicular neoplasm or suspicious for follicular neoplasm
SM: Suspicious for malignancy
ITNs: Indeterminate thyroid nodules
GEC: Gene expression classifier
PLR: Positive likelihood ratio
NLR: Negative likelihood ratio
ATA: American Thyroid Association
TI-RADS: The thyroid imaging reporting and data system.
